# Supplementary material for: Modeling glioblastoma heterogeneity as a dynamic network of cell states
Source: Mol Syst Biol. 2021 Sep 16;17(9):e10105. doi: 10.15252/msb.202010105 (PMC8444284; doi:10.15252/msb.202010105)
Supplement: Supplementary file 6 — Source Data for Figure 5 [file MSB-17-e10105-s004.zip › Figure5A_sourcedata/GSEA_3071/hallmarks_stateIV.GseaPreranked.1623742551702/HALLMARK_REACTIVE_OXYGEN_SPECIES_PATHWAY.html]

Details for gene set HALLMARK\_REACTIVE\_OXYGEN\_SPECIES\_PATHWAY[GSEA]

|  || Dataset | state53071 |
| Phenotype | NoPhenotypeAvailable |
| Upregulated in class | na\_neg |
| GeneSet | HALLMARK\_REACTIVE\_OXYGEN\_SPECIES\_PATHWAY |
| Enrichment Score (ES) | -0.5387219 |
| Normalized Enrichment Score (NES) | -2.0351243 |
| Nominal p-value | 0.0018382353 |
| FDR q-value | 0.0012934743 |
| FWER p-Value | 0.011 |
Table: GSEA Results Summary

  

Fig 1: Enrichment plot: HALLMARK\_REACTIVE\_OXYGEN\_SPECIES\_PATHWAY      
 Profile of the Running ES Score & Positions of GeneSet Members on the Rank Ordered List

  

| PROBE | GENE SYMBOL | GENE\_TITLE | RANK IN GENE LIST | RANK METRIC SCORE | RUNNING ES | CORE ENRICHMENT || 1 | PRDX2 |  |  | 143 | 1.387 | 0.0351 | No |
| 2 | GPX4 |  |  | 480 | 0.831 | 0.0086 | No |
| 3 | NDUFB4 |  |  | 999 | 0.522 | -0.0663 | No |
| 4 | PRNP |  |  | 1110 | 0.488 | -0.0653 | No |
| 5 | MSRA |  |  | 2176 | 0.271 | -0.2554 | No |
| 6 | CDKN2D |  |  | 2615 | -0.284 | -0.3258 | No |
| 7 | GSR |  |  | 3021 | -0.335 | -0.3877 | No |
| 8 | MBP |  |  | 3231 | -0.365 | -0.4110 | No |
| 9 | TXNRD2 |  |  | 3394 | -0.390 | -0.4243 | No |
| 10 | NDUFA6 |  |  | 3511 | -0.412 | -0.4278 | No |
| 11 | PRDX6 |  |  | 4001 | -0.511 | -0.4977 | No |
| 12 | SRXN1 |  |  | 4150 | -0.555 | -0.5009 | No |
| 13 | FTL |  |  | 4350 | -0.615 | -0.5111 | Yes |
| 14 | GLRX |  |  | 4374 | -0.626 | -0.4874 | Yes |
| 15 | TXNRD1 |  |  | 4382 | -0.629 | -0.4605 | Yes |
| 16 | G6PD |  |  | 4698 | -0.796 | -0.4845 | Yes |
| 17 | PRDX1 |  |  | 4783 | -0.848 | -0.4624 | Yes |
| 18 | PRDX4 |  |  | 4889 | -0.954 | -0.4395 | Yes |
| 19 | GLRX2 |  |  | 4907 | -0.973 | -0.3991 | Yes |
| 20 | PFKP |  |  | 5042 | -1.173 | -0.3719 | Yes |
| 21 | GCLM |  |  | 5077 | -1.235 | -0.3229 | Yes |
| 22 | TXN |  |  | 5080 | -1.246 | -0.2673 | Yes |
| 23 | LSP1 |  |  | 5095 | -1.278 | -0.2126 | Yes |
| 24 | PDLIM1 |  |  | 5142 | -1.392 | -0.1588 | Yes |
| 25 | NQO1 |  |  | 5178 | -1.556 | -0.0956 | Yes |
| 26 | MGST1 |  |  | 5275 | -2.599 | 0.0028 | Yes |
Table: GSEA details [plain text format]

  

Fig 2: HALLMARK\_REACTIVE\_OXYGEN\_SPECIES\_PATHWAY: Random ES distribution      
 Gene set null distribution of ES for **HALLMARK\_REACTIVE\_OXYGEN\_SPECIES\_PATHWAY**
